# Detrimental association between betel nut chewing and colorectal polyps in adult populations

**DOI:** 10.1371/journal.pone.0206383

**Published:** 2018-10-25

**Authors:** Yuan-Yuei Chen, Wen-Hui Fang, Chung-Ching Wang, Tung-Wei Kao, Yaw-Wen Chang, Hui-Fang Yang, Chen-Jung Wu, Yu-Shan Sun, Wei-Liang Chen

**Affiliations:** 1 Department of Internal Medicine, Tri-Service General Hospital Songshan Branch and School of Medicine, National Defense Medical Center, Taipei, Taiwan, Republic of China; 2 Division of Family Medicine, Department of Family and Community Medicine, Tri-Service General Hospital and School of Medicine, National Defense Medical Center, Taipei, Taiwan, Republic of China; 3 Health Management Center, Department of Family and Community Medicine, Tri-Service General Hospital, National Defense Medical Center, Taipei, Taiwan, Republic of China; 4 Division of Geriatric Medicine, Department of Family and Community Medicine, Tri-Service General Hospital and School of Medicine, National Defense Medical Center, Taipei, Taiwan, Republic of China; 5 Graduate Institute of Clinical Medical, College of Medicine, National Taiwan University, Taipei, Taiwan, Republic of China; 6 Division of Family Medicine, Department of Community Medicine, Taoyuan Armed Forces General Hospital, Taoyuan, Taiwan, Republic of China; University of South Alabama Mitchell Cancer Institute, UNITED STATES

## Abstract

Adverse systemic effect caused by betel nut had been reported for decades. Our aim was to determine whether betel nut had detrimental impact on the development of colorectal polyps in general population. Participants who attended health examinations at the Tri-Service General Hospital (TSGH) from 2010 to 2016 were included in the study. The habit of betel nut chewing was obtained from a self-reported questionnaire. Colorectal polyps were diagnosed by colonoscopies operated by experienced physicians. A logistic regression model was used for the association between betel nut chewing with the presence of colorectal polyps. After adjustment for pertinent information such as age, gender, biochemistry data and personal history, the odd ratios (ORs) of colorectal polyps among betel nut chewers was 1.49 (95%CI: 1.14–1.94). Besides, betel nut chewers in the higher percentage body fat (PBF) group had higher risk for developing colorectal polyps with ORs of 2.07 (95%CI:1.23–3.47). Subjects with habit of betel nut chewing were associated with an increased risk of colorectal polyps in Taiwanese general population. Screening for betel nut chewing history and encouraging cessation might offer improved quality of life. A further research for this association was warranted.

## Introduction

Betel nut is regarded as one of the frequently used addictive substance worldwide and betel nut chewers were prevalent in Asian countries more than 10% world’s population[1, 2]. Traditionally, people often chew betel nuts in combination with additional elements such as *P*. *betle* and lime in Taiwan[3]. Arecal alkaloid, which is the main components of betel nut, is absorbed in human body during chewing[2]. Arecoline, arecaidine, guvacoline and guvacine were the main alkaloids of betel nut and caused several systemic effects including nervous, cardiovascular, gastrointestinal and endocrine system[4–7]. Growing evidence had already proposed that these components contributed to various systemic diseases including obesity, diabetes mellitus, and metabolic syndrome[8–10]. Additionally, betel nut chewing could increase the risk of cardiovascular diseases and development of oral, esophagus and hepatocellular carcinoma[11–13].

In Taiwan, the incidence rates of colon and rectum cancer were 9299/10^6^ and 5559/10^6^, respectively in 2012[14]. According to the statistical data of Taiwan Health Promotion Administration of the Ministry of Health and Welfare in 2016[15], the mortality cases of colorectal cancer were 5772 and the mortality rates were 14.6/10^6^. The prevalence of colorectal polyps in asymptomatic subjects was 27.4%[16]. Accumulated literatures indicated that most colorectal cancers were originated from a precursor benign polyp[17]. Race, gender, obesity and metabolic syndrome were common factors that increased risk of developing colorectal polyps[18, 19]. Besides, lifestyle including cigarette smoking, alcoholic consumption, and lack of physical activity were also reported to accelerate the neoplastic process[20–22].

To date, the association of consumption of betel nut and colorectal polyps had not been reported in previous study. The main goal of our study was to determine the effect of betel nut on the progress of colorectal polyps by using a large population-based cross-sectional analysis in a general population in Taiwan.

## Methods

### Study design and participants selection

**Fig 1:**
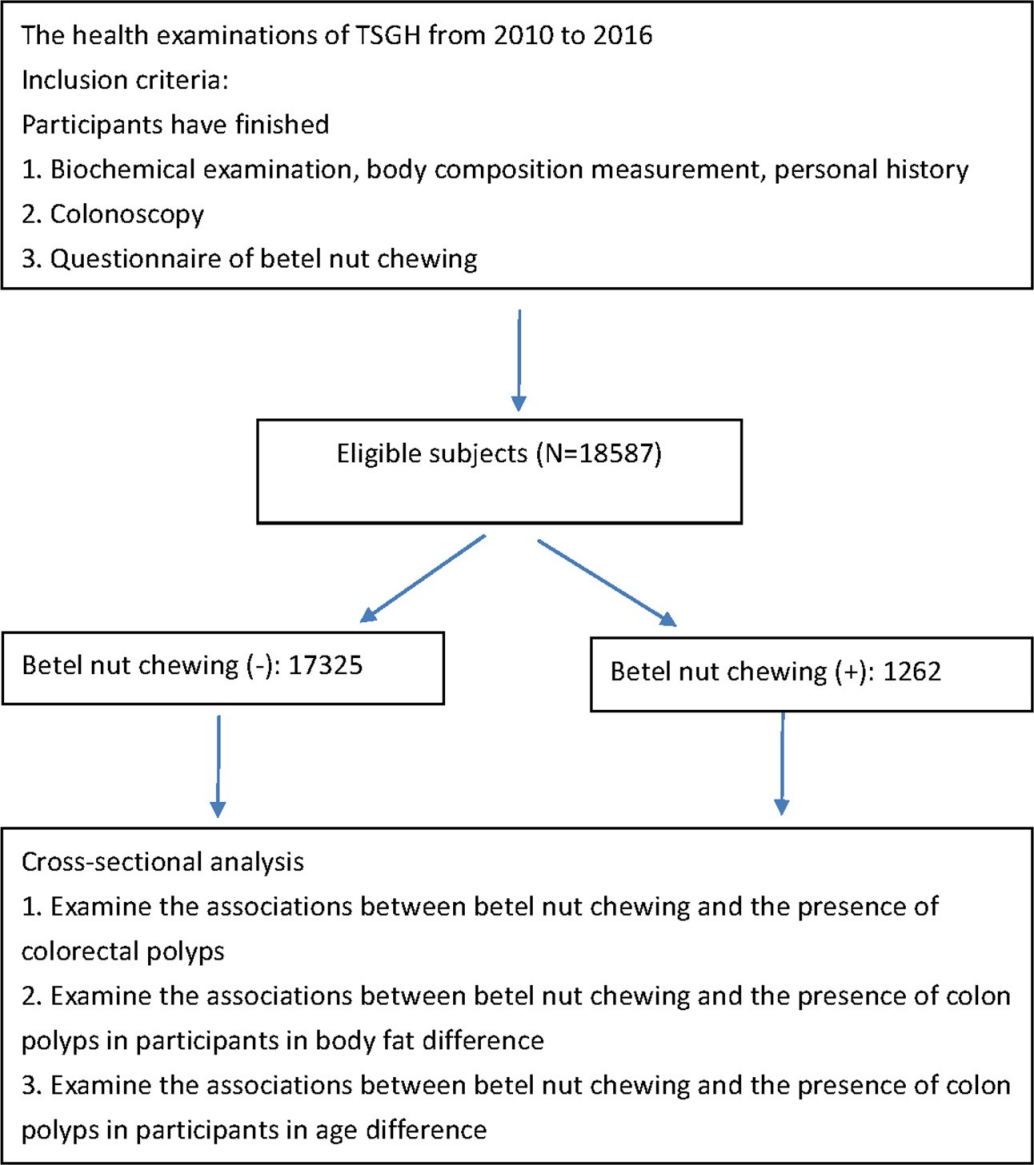
Flow chart which represented the steps of analysis performed in the study.

The health examinations at Tri-Service General Hospital (TSGH) were consisted of comprehensive medical records including laboratory biochemistry data, body composition measurement and self-reported personal history. 69226 participants aged more than 20 years underwent health examinations from 2010 to 2016. All protocol was approved by the institutional review board of the Tri-Service General Hospital (TSGH) with patient written consent given. The study was conducted according to the Helsinki Declaration. The TSGH Institutional Review Board waived the need to obtain individual informed consent because the data were analyzed anonymously. Based on the inclusion criteria presented in Fig 1, participants who finished biochemical examination, body composition measurement, colonoscopy, and questionnaire of betel nut chewing were included. 18587 eligible individuals with and without habits of betel nut chewing were analyzed in the next step. First, we explored whether betel nut chewing was associated with the risk of developing colorectal polyps. Next, we investigated the relationship between betel-chewing behavior and the presence of colorectal polyps in body fat and age difference.

### Diagnosis of colorectal polyps

Colonoscopic examinations were operated by trained physicians. Digital of rectum before the endoscope was the routine in the examination. Participants took a laxative on the night before the examination and in the morning of the day for colonoscopy. All visualized lesions were biopsied and histologically assessed by experienced pathologists. For the purpose of our analysis, participants were categorized into two groups according to the presence of colorectal polyps.

### Measurement of body composition

Percentage body fat (PBF) is measured by bioelectrical impedance analysis (BIA) (InBody720, Biospace, Inc., Cerritos, CA, USA), a useful method for assessing body composition[23]. The procedure of BIA is simple and noninvasive, and the results were reproducible and rapidly obtained.

### Covariates measurement

The pertinent information of study sample is collected by a self-report questionnaire consisted of age, sex, history of cigarette smoking and alcoholic consumption. Body mass index (BMI) is defined as the body weight divided by the square of the body height (kg/m^2^) of a participant. Latex nephelometry is used for analyzed the concentrations of highly sensitive C-reactive protein (hsCRP). Serum low-density lipoprotein cholesterol (LDL-C) is quantified by an enzymatic colorimetric method.

### Statistical analysis

Multivariable models were adjusted as follows: Model 1 was adjusted as for age and gender. Model 2 was adjusted as for model 1 plus proteinuria, LDL-C, uric acid (UA), aspartate aminotransferase (AST), creatinine (Cr), hsCRP. Model 3 was adjusted as for Model 2 plus history of smoking and alcoholic drinking. All statistical analyses in the present study were analyzed by the SPSS Statistics software package (version 18.0, SPSS Inc., Chicago, IL, USA) for Windows. A two-sided *P*-value of < 0.05 was considered statistically significant. Multiple logistic regression was performed for the association of betel-chewing behavior on the presence of colorectal polyps.

## Results

### Characteristics of study sample

All characteristics of the study sample were listed in Table 1. The mean age of the non-users and betel nut chewers was 46.04±13.11 and 46.89±11.54 years old. Betel nut chewers had significantly higher BMI, LDL-C, UA, Cr, AST, albumin, and hsCRP than non-users.

**Table 1:** Characteristics of study sample with or without betel nut chewing.

Variables	Betel nut chewing (-) (N = 17325)	Betel nut chewing (+) (N = 1262)	P Value
**Continuous Variables, mean (SD)**			
** Age (years)**	46.04 (13.11)	46.89 (11.54)	<0.05
** BMI (kg/m**^**2**^**)**	24.02 (3.72)	25.97 (3.62)	<0.01
** LDL-C (mg/dL)**	117.44 (31.86)	119.83 (35.16)	<0.05
** Uric acid (mg/dL)**	5.75 (1.49)	6.61 (1.52)	<0.01
** Creatinine (mg/dL)**	0.84 (0.31)	0.96 (0.26)	<0.01
** AST (U/L)**	21.05 (11.66)	23.07 (11.88)	<0.01
** Albumin (g/dL)**	4.46 (0.28)	4.49 (0.28)	<0.01
** hsCRP (mg/dL)**	0.22 (0.46)	0.32 (0.68)	<0.01
** TSH (IU/mL)**	2.25 (1.62)	2.18 (2.19)	0.18
**Category Variables, (%)**			
** Gender (male)**	9002 (58.2)	824 (98.3)	<0.01
** Proteinuria**	4297 (27.3)	311 (34.7)	<0.01
** Smoking**	4499 (25.6)	877 (89.9)	<0.01
** Drinking**	7921 (45.1)	827 (85.5)	<0.01

**BMI, body mass index; LDL-C, low density lipoprotein cholesterol; UA, uric acid; Cr, creatinine; AST, aspartate aminotransferase; hsCRP, high sensitive C-reactive protein; TSH, thyroid stimulating hormone**.

### Betel-chewing behavior and colorectal polyps

As shown in Table 2, the univariate logistic regression model revealed the odds ratios (ORs) of each covariable for the presence of colorectal polyps. The ORs of betel nut chewing were 2.39 (confidence interval (CI): 1.97–2.89). All covariables were significantly associated with the presence of colorectal polyps, except albumin.

**Table 2:** Univariate regression analyses for the presence of colorectal polyps.

Variables	OR (95%CI)	P-value
**Continuous Variables**		
** Age (years)**	1.06 (1.05–1.06)	<0.01
** BMI (kg/m**^**2**^**)**	1.07 (1.05–1.08)	<0.01
** PBF**	1.02 (1.01–1.02)	<0.01
** LDL-C (mg/dL)**	1.01 (1.01–1.01)	<0.01
** Uric acid (mg/dL)**	1.11 (1.07–1.15)	<0.01
** Creatinine (mg/dL)**	1.27 (1.12–1.44)	<0.01
** AST (U/L)**	1.01 (1.01–1.02)	<0.01
** Albumin (g/dL)**	0.92 (0.75–1.13)	0.44
** hsCRP (mg/dL)**	1.16 (1.05–1.29)	<0.01
**Category Variables**		
** Betel nut chewing**	2.39 (1.97–2.89)	<0.01
** Gender**	1.76 (1.53–2.02)	<0.01
** Proteinuria**	1.48 (1.30–1.67)	<0.01
** Smoking**	2.59 (2.31–2.90)	<0.01
** Drinking**	1.48 (1.32–1.66)	<0.01

The association of betel nut chewing on the presence of colorectal polyps was analyzed by a multiple logistic regression model shown in Table 3. The ORs in the Model 1 for colorectal polyps among subjects with betel nut chewing was 2.11 (95% CI:1.63–2.72). Slight attenuate in this relationship was noted but remained significant after adjustment for biochemical data and personal history in Model 2 and Model 3 with ORs of 2.00 (95%CI: 1.55–2.59) and 1.49 (95%CI: 1.14–1.94), respectively.

**Table 3:** Multivariate regression analyses for association between betel nut chewing and the presence of colon polyps.

Models	Model ^a^ 1 OR (95% CI)	*P* Value	^*b*^*R*^2^	Model ^a^ 2 OR (95% CI)	*P* Value	^*b*^*R*^2^	Model ^a^ 3 OR (95% CI)	*P* Value	^*b*^*R*^2^
**Variables**	**Colorectal polyps**								
**Betel nut chewing** **(N = 1262)**	2.11 (1.63–2.72)	<0.01	0.11	2.00 (1.55–2.59)	<0.01	0.13	1.49 (1.14–1.94)	<0.01	0.15

^a^ Adjusted covariates:

Model 1 = age + gender

Model 2 = Model 1 + proteinuria, LDL-C, UA, Cr AST, hsCRP

Model 3 = Model 2 + history of smoking, drinking

^b^ Nagelkerke R squared

### Betel nut chewing and colorectal polyps in different body fat percentage

In Table 4, we categorized betel nut chewers as PBF tertiles. After fully adjusting for pertinent covariables, betel nut chewing was significantly associated with the presence of colorectal polyps in T1 and T3 with ORs of 1.58 (95%CI: 1.00–2.49) and 2.07 (95%CI: 1.23–3.47), respectively. However, no significant association between T2 and the presence of colorectal polyps in the fully-adjusted model.

**Table 4:** Association between betel nut chewing and the presence of colorectal polyps categorized by the tertiles of PBF.

Models	PBF Group	Model ^a^ 1 OR (95% CI)	*P* Value	^*b*^*R*^2^	Model ^a^ 2 OR (95% CI)	*P* Value	^*b*^*R*^2^	Model ^a^ 3 OR (95% CI)	*P* Value	^*b*^*R*^2^
**Variables**	**Colorectal polyps**									
**Betel nut chewing**	Tertile 1	2.21 (1.43–3.42)	<0.01	0.09	2.21 (1.42–3.43)	<0.01	0.11	1.58 (1.00–2.49)	<0.05	0.13
	Tertile 2	1.29 (0.83–1.99)	0.26	0.10	1.25 (0.80–1.94)	0.33	0.12	0.95 (0.60–1.50)	0.83	0.13
	Tertile 3	2.56 (1.55–4.21)	<0.01	0.10	2.51 (1.52–4.14)	<0.01	0.10	2.07 (1.23–3.47)	<0.01	0.11

^a^ Adjusted covariates:

Model 1 = age + gender

Model 2 = Model 1 + proteinuria, LDL-C, UA, Cr AST, hsCRP

Model 3 = Model 2 + history of smoking, drinking

^b^ Nagelkerke R squared.

### Different age groups in association between betel nut chewing and the presence of colorectal polyps

As shown in Table 5, we categorized betel nut chewers as different age groups (20–29 years, 30–39 years, 40–49 years, 50–59 years, and ≧60 years). After fully adjusting for covariables, betel nut chewing was significantly associated with the presence of colorectal polyps in 40–49 and ≧60 age groups with ORs of 1.77 (95%CI: 1.06–2.95) and 1.84 (95%CI: 1.00–3.38), respectively.

**Table 5:** Different age groups in association between betel nut chewing and the presence of colon polyps.

Models	Age Group	Model ^a^ 1 OR (95% CI)	*P* Value	^*b*^*R*^*2*^	Model ^a^ 2 OR (95% CI)	*P* Value	^*b*^*R*^*2*^	Model ^a^ 3 OR (95% CI)	*P* Value	^*b*^*R*^*2*^
**Variables**	**Colorectal polyps**									
**Betel nut chewing**	20–29	Reference	-	0.10	Reference	-	0.34	Reference	-	0.36
	30–39	2.97 (1.41–6.23)	<0.01	0.05	2.61 (1.21–5.62)	<0.05	0.09	1.99 (0.89–4.45)	0.09	0.10
	40–49	2.66 (1.65–4.30)	<0.01	0.05	2.49 (1.52–4.088)	<0.01	0.08	1.77 (1.06–2.95)	<0.05	0.10
	50–59	1.40 (0.93–2.11)	0.11	0.05	1.33 (0.88–2.01)	0.18	0.07	1.06 (0.69–1.62)	0.79	0.09
	≧60	2.27 (1.27–4.04)	<0.01	0.06	2.31 (1.28–4.17)	<0.01	0.08	1.84 (1.00–3.38)	<0.05	0.09

^a^ Adjusted covariates:

Model 1 = age + gender

Model 2 = Model 1 + proteinuria, LDL-C, UA, Cr AST, hsCRP

Model 3 = Model 2 + history of smoking, drinking

^b^ Nagelkerke R squared

## Discussion

Our findings highlighted the detrimental association between betel nut chewing and colorectal polyps in a cross-sectional analysis. Interestingly, betel nut chewers with increased PBF were positively associated with the risk of colorectal polyps, but those with reduced PBF were not. To the best of our knowledge, our study was the first to examine the impact of betel nut chewing on the process of colorectal polyps and observed the opposite findings in difference of body fat distribution.

To date, no published literature had addressed the relationship between betel nut chewing and colorectal polyps. There were several factors that increase risk of colorectal polyps including race, gender, smoking, and obesity[18]. The key factor jointed the obesity and the development of colorectal polyps was insulin resistance[24]. It contributed to several metabolic abnormalities such as excess glucose levels and dyslipidemia leading to alterations in cell signaling and oxidative stress[25]. Trabulo et al. had demonstrated that metabolic syndrome was significantly associated with the development of colorectal adenomas and cancer[26]. A meta-analysis had supported the harmful impact of metabolic syndrome in process of colorectal neoplasms[19]. Arecoline, arecaidine, guvacoline and guvacine were the main alkaloids of betel nut and caused several systemic effects including nervous, cardiovascular, gastrointestinal and endocrine system[4–7]. The metabolism of arecoline was hydrolysis to arecaidine and N-oxidation combined with double bond reduction of the arecaidine[27]. Chung et al. had reported that chronic betel nut chewing was an important factor for developing metabolic syndrome[28]. In a population-based prospective study, betel nut chewing was significant associated with metabolic syndrome and it had increased risks of metabolic syndrome component such as central obesity, hypertension, hyperglycemia and dyslipidemia[10]. Taking above researches together, metabolic dysfunction might be a potential mechanism between betel nut chewing and the presence of colorectal polyps, which was the main finding in our study.

Although the underlying mechanism of how betel nut causing metabolic syndrome remained unclear, various possible pathways had been suggested earlier. Betel nut chewing was considered to be related to increased levels of prostanoids, interleukin-6 (IL-6), and tumor necrosis factor-α (TNF-α)[29]. These inflammatory mediators induced a state of low-grade chronic inflammation and were associated with insulin resistance and metabolic alternation[30, 31]. Hsu et al. had indicated that arecoline led to the development of insulin resistance and metabolic diseases by inhibiting adipogenic differentiation such as inducing adenylyl cyclase-dependent lipolysis and interfering insulin-induced glucose uptake[32]. One of the potential mechanisms of the interaction between metabolic syndrome with colonic neoplasms was oxidative stress. DNA damage caused by increased reactive oxygen species was found in those with metabolic syndrome and thus placed subjects at risk for neoplasm process[33]. Another plausible mechanisms was dysregulation of cytokines and growth signals including insulin growth factor-1 (IGF-1), insulin and adipokines, which might contribute to cancer-related process[34–36]. Inflammatory cytokines such as IL-6 and TNF-α were responsible to activation of signal transducers and activators of transcription factors[35, 37]. IGF-1 and hyperinsulinemia were reported to induce PI3K/Akt activity that regulated downstream targets.[38]. All these molecules were responsible for cell proliferation, tumor growth, and changes from normal colonic mucosa to adenoma and adenocarcinoma[39].

Obesity was considered to have adverse impact on the development of colorectal neoplasms and visceral obesity was associated with higher risk for colorectal cancer than BMI[40, 41]. Accumulation of visceral adipose tissue was associated with insulin resistance, increased circulating levels of leptin and reduced adipokine[42]. Epidemiological and clinical researches had indicated that altered concentrations of leptin and adipokine might lead to the presence of colorectal adenoma and carcinoma[43, 44]. Above studies could support our finding that betel nut chewers in the higher PBF group had increased risk of developing colorectal polyps.

Despite the strength of a large population-based design, our study still had some potential limitations. First, casual inference between betel nut chewing and colorectal polyps was no assessible in the present study because it was a cross-sectional design. A longitudinal survey was suggested to be examined in further studies. Second, the health examinations only recorded the personal history of betel nut chewing that were lacking detailed information about how many betel nut uses per day and the duration of usage. Thus, the cumulative exposure of betel nut could not be accessed in the current study. Next, study sample was composed of healthy general population who underwent health examinations but not from nationally representative individuals. External validation was necessary in further studies. Last, all results of colonoscopic examinations in the present study were obtained from health examinations at the Health Management Center of TSGH. The different types of colorectal polyps are not available in our dataset.

## Conclusion

While the systemic effect of betel nut chewing had been known for decades, detrimental impact on development of colorectal polyps was first reported among a general population in Taiwan of our study. Screening personal history of betel nut chewing in clinical practices and encouraging cessation might offer an improved quality of life for individuals who were at risk of systemic diseases by betel nut use. Furthermore, attention on this risk factor through public health programs and further investigation in association between betel nut chewing and colorectal diseases were necessary.
